# From Injury to Full Recovery: Monitoring Patient Progress Through Advanced Sensor and Motion Capture Technology

**DOI:** 10.3390/s25133853

**Published:** 2025-06-20

**Authors:** Annchristin Andres, Michael Roland, Marcel Orth, Stefan Diebels

**Affiliations:** 1 Applied Mechanics, Saarland University, 66123 Saarbrücken, Germany; 2 Department of Trauma, Hand and Reconstruction Surgery, Saarland University Hospital, 66424 Homburg, Germany

**Keywords:** digital mobility outcome, wearables, patient monitoring, rehabilitation, fracture healing

## Abstract

Background: Advanced sensor insoles and motion capture technology can significantly enhance the monitoring of rehabilitation progress for patients with distal tibial fractures. This study leverages the potential of these innovative tools to provide a more comprehensive assessment of a patient’s gait and weight-bearing capacity following surgical intervention, thereby offering the possibility of improved patient outcomes. Methods: A patient who underwent distal medial tibial plating surgery in August 2023 and subsequently required revision surgery due to implant failure, involving plate removal and the insertion of an intramedullary nail in December 2023, was meticulously monitored over a 12-week period. Initial assessments in November 2023 revealed pain upon full weight-bearing without crutches. Following the revision, precise weekly measurements were taken, starting two days after surgery, which instilled confidence in accurately tracking the patient’s progress from initial crutch-assisted walking to full recovery. The monitoring tools included insoles, hand pads for force absorption of the crutches, and a motion capture system. The patient was accompanied throughout all steps of his daily life. Objectives: The study aimed to evaluate the hypothesis that the approximation and formation of a healthy gait curve are decisive tools for monitoring healing. Specifically, it investigated whether cadence, imbalance factors, and ground reaction forces could be significant indicators of healing status and potential disorders. Results: The gait parameters, cadence, factor of imbalance ground reaction forces, and the temporal progression of kinematic parameters significantly correlate with the patient’s recovery trajectory. These metrics enable the early identification of deviations from expected healing patterns, facilitating timely interventions and underscoring the transformative potential of these technologies in patient care. Conclusions: Integrating sensor insoles and motion capture technology offers a promising approach for monitoring the recovery process in patients with distal tibial fractures. This method provides valuable insights into the patient’s healing status, potentially predicting and addressing healing disorders more effectively. Future studies are recommended to validate these findings in a larger cohort and explore the potential integration of these technologies into clinical practice.

## 1. Introduction

Gait analysis has become an important parameter for monitoring the healing process of injuries and various medical conditions [1]. It has also been useful for monitoring improvements in spatiotemporal gait parameters, kinematics, kinetics, and pedography throughout the healing process in lower leg fractures [2].

Wearable sensors, such as gait monitoring insoles, have been used to monitor patient behavior during rehabilitation [3]. These sensors track metrics like step count, walking time, cadence, and body weight per step, offering insights that influence rehabilitation outcomes [4]. Additionally, insole-force sensors measure loading on the lower extremities in patients with pelvic fragility fractures [4]. This study found differences in loading between the affected and unaffected limbs, suggesting that using insole-force sensors may improve treatment choices and timing [5]. Telemetric gait analysis insoles track postoperative fracture rehabilitation by analyzing ground reaction forces [6]. Combined with a mobile application and a convolutional neural network, these insoles can automate the monitoring of patient progress and identify bone healing disorders such as non-unions.

Recent advancements in sensor technology and motion capture systems have significantly transformed the rehabilitation landscape, particularly in the monitoring and management of partial weight-bearing (PWB) in individuals recovering from lower limb surgeries, injuries, or other medical conditions [7]. The critical role of PWB in rehabilitation involves limiting the amount of weight placed on the affected limb to prevent stress and pain, promote healing, and ensure a successful recovery by reducing the risk of re-injury and facilitating a smooth transition to full weight-bearing throughout the healing course. Integrating sensor insoles and motion-capturing systems has emerged as a breakthrough in enhancing the monitoring and guidance of PWB [8,9].

Sensor insoles (Moticon^TM^, ReGo AG, Munich, Germany), designed to provide real-time feedback on foot pressure distribution, enable precise monitoring of how much weight a patient places on different aspects of the affected limb [9,10]. Likewise, the motion-capturing system from Xsens^TM^ (Movella Technology B.V., Enschede, The Netherlands) delivers an in-depth analysis of the patient’s movement patterns, capturing a wide array of data points that facilitate monitoring progress and adjusting rehabilitation strategies. Xsens^TM^ technology allows for accurate assessments of gait, balance, and other functional movements, providing invaluable insights into a patient’s recovery trajectory. Recent studies’ contributions underscore the advancements and applications of these technologies in rehabilitation. For instance, Horenstein and colleagues [11,12] validated wireless inertial sensors for hip joint motion, demonstrating that most angles measured with the sensor-based system were within 6° of those measured with a traditional camera motion capture system.

By combining the capabilities of advanced sensor insoles and motion-capturing systems, healthcare professionals can comprehensively understand a patient’s prescribed PWB adherence and overall movement health. This integrated approach enables a more tailored and effective rehabilitation process, leveraging the power of technology to facilitate better outcomes for patients navigating the complexities of PWB. These technological advancements, supported by a growing body of research, underscore the potential of sensor and motion capture technologies in revolutionizing rehabilitation practices and improving patient care. However, it remains unclear to what extent a thoroughly analyzed patient’s gait curve over an entire healing cycle can be a critical indicator of successful bone healing. The study hypothesizes that approximating and stabilizing a patient’s gait curve toward a normative healthy gait pattern are essential indicators of successful healing. We used metrics such as cadence, imbalance factor, and GRFs over time to assess the healing process and identify potential complications or delayed recovery.

## 2. Methods

Different activities that reflect the patient’s daily life are part of a measurement workflow [13]. These include walking twice with crutches, climbing stairs, performing a sit-to-stand-to-sit cycle, and dorsiflexion and plantarflexion of his injured foot. The various exercises were measured simultaneously using sensor insoles and a motion-capturing system for activities such as climbing stairs, sitting down, and standing up, as well as navigating stairs in the hallway and using couches or dining room chairs in the patient’s apartment. The supplementary material provides an example for the knee joint angle (see Figure S3) and the ground reaction force (see Figure S4) during walking. The purpose of the whole exercise is to represent his everyday situation as accurately as possible and the effect of these stresses. For example, stair climbing was essential in his daily living routine as his apartment is on the second floor, and he had to walk up and down several times a day from the outset. Table 1 details any deviations from this workflow.

### 2.1. Kinetic Gait Analysis

Measuring soles dynamically measure the ground reaction force and pressure distribution in the foot, making them essential for analyzing load scenarios and gait patterns. These self-sufficient measuring soles record with a sampling rate of 100 Hz. Each sole comprises sixteen pressure sensors, an acceleration sensor, and a temperature sensor. Figure S1 in the supplementary material provides a detailed image of the insoles. In kinetic gait analysis, particularly in observing the healing process by increasing the load-bearing capacity of the fractured leg, the ground reaction force plays a crucial yet straightforward interpretative role as per Newton’s third law, which states that action equals reaction. The force that the ground exerts on a body in contact with it is a vector quantity representing the reaction force exerted by the ground on a person or object while standing, walking, or running. For the evaluation, the cadence (1), that is, the number of steps taken per minute, is considered on the one hand. The imbalance factor (2) more accurately represents the ground reaction force ratio between the healthy left leg and the fractured right leg. It quantifies asymmetry in percent between the left (healthy) leg (GRF_left) and right (injured) leg (GRF_right) ground reaction force.

(1) $$Cadence=\frac{Number\;of\;Steps}{Time\;[minute]}$$

(2) $$Factor\;of\;Imbalance=\frac{\left|\left(GRF\_left-GRF\_right\right)\right|}{GRF\_left}\;*\;100$$

### 2.2. Kinematic Gait Analysis

The Xsens^TM^ motion capture system “Awinda” tracks movements to create a full-body motion image, enabling immediate graphical output. Additionally, the system can determine joint angle data, center of gravity, and other sensor information. It comprises 17 inertial measurement units (IMUs) strategically attached to important body parts using straps. The sensor placement is bilateral on the thighs, shanks, feet, shoulders, hands, and lower and upper arms, as well as on the pelvis, trunk, and head. The 17 wireless inertial measurement units contain a 3D magnetometer to measure the 3D geomagnetic field, a 3D velocity gyroscope to measure the 3D angular velocity, a linear accelerometer to measure the 3D acceleration, and a barometer to measure the atmospheric pressure. The system recorded full-body kinematics at 60 Hz. Figure S2 in the supplementary material shows the complete setup of the system. The measured values from the lower body motion trackers—specifically those on the right and left thighs, lower legs, and feet—played a crucial role in analyzing this patient’s gait. Due to the existing tibia fracture, examining the knee joint angle in greater detail was essential. The study examined the angle of the knee joint during both the stance and swing phases of walking. While extensive research has already been conducted on healthy individuals as evidenced in various literature sources [14,15,16,17], our studies, presented in the supplementary material, offer additional validation. The knee joint angle is a three-dimensional Euler angle derived from the transformation between the thigh and shank coordinate frames. In Xsens^TM^, it is calculated based on positional data from the motion tracker on the thigh and the tracker on the lower leg. Our analysis focuses specifically on knee flexion and extension as these movements exhibit the most significant variations in response to the injury [18,19]. For the subsequent evaluations, the premise was also to take simple values with a relatively high significance, which do not require much prior knowledge for analysis. Acceleration is the first value taken directly from the measurement data. The acceleration variation between the right and left legs is a percentage for better visualization. The recorded measurement data allows easy retrieval of these values. The knee joint angle is subdivided into stance and swing phases as it reaches two maxima during a healthy stride. The first has up to 15 degrees of flexion in the loading response and thus falls in the middle of the stance phase, while the second has up to 60 degrees of flexion in the initial swing phase. Therefore, the deviations and approximations during the recovery phase are of interest. The analysis also includes the stride length, which is the distance between the position of a heel at the foot strike and that of the same heel at its next foot strike from the start to the end of a gait cycle.

## 3. Patient

**Figure 1:**
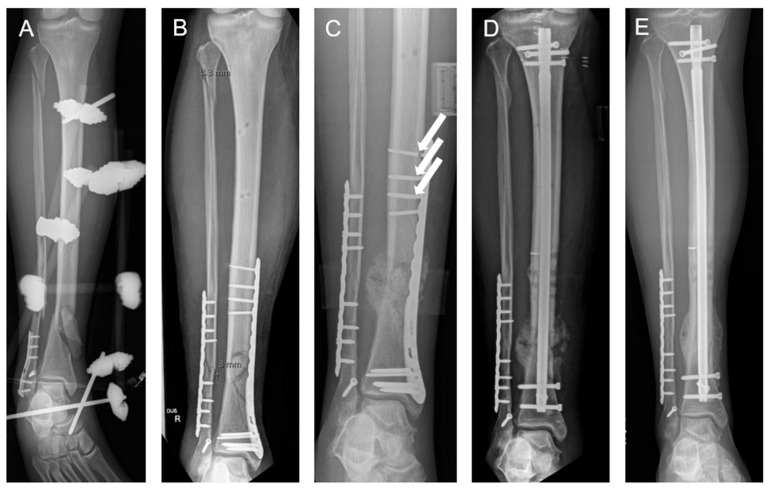
A 29-year-old male patient suffering from a lower leg fracture after a previous and healed ankle fracture in the past (**A**). The patient was initially treated by plating osteosynthesis of the tibia and fibula (**B**). At 4 months after surgery, the patient showed an implant failure ((**C**); arrows) and signs of a hypertrophic non-union as a disturbed bone healing course. After revision surgery (**D**), the patient underwent a clinically uneventful course and demonstrated full bone healing 6 months post-revision surgery (**E**).

A 12-week longitudinal monitoring study followed a patient who sustained a distal tibial fracture in August 2023 and received fixation with a distal medial tibial locking compression plate (LCP) (Figure 1). In November 2023, the patient returned for biomechanical assessment and underwent initial gait measurements using instrumented insoles and Xsens™ motion capture technology. At that time, the patient reported pain with every step but was walking independently with full weight-bearing and without crutches. In December 2023, surgeons performed a revision surgery, replacing the LCP with an intramedullary nail. Gait measurements began two days after the operation. Over the following 12 weeks, the system recorded the patient’s gait parameters weekly using Xsens™ and instrumented insoles, tracking progress through to full recovery.

## 4. Results

**Figure 2:**
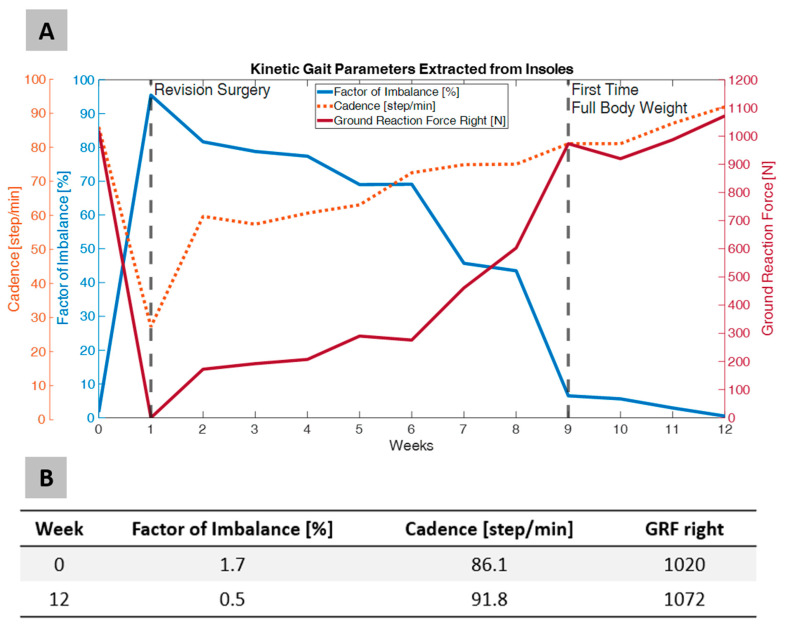
Temporal evolution of kinetic parameters, including a factor of imbalance in percentage, cadence in steps per minute, and ground reaction force of the fractured right leg in Newtons, over a 12-week recovery period following revision surgery (**A**), and exact values for week 0 in grey and week 12 in white (**B**).

The monitoring focused on key parameters, including cadence, ground reaction forces (GRFs), and imbalance factors, to assess changes in the patient’s gait pattern and its approximation to a healthy gait curve (Figure 2). The factor of imbalance initially spikes (near 100%) due to severe asymmetry in gait caused by the fracture and surgery. Over time, there is a gradual reduction, approaching near-normal levels (~10%) by week 12, indicating improved symmetry in weight distribution between the legs. Cadence (steps per minute) begins relatively low, around 30 steps per minute, and steadily increases throughout the recovery period, plateauing at approximately 60–65 steps per minute after week 8. This trend reflects an improved gait rhythm and increased confidence in walking. The ground reaction force on the right leg starts near zero, indicating minimal weight-bearing capacity. After initiating full-body weight-bearing at week 6, the ground reaction force significantly increases, exceeding 1000 N by week 12.

This trend suggests strengthening of the right leg and the restoration of load-bearing functionality. Over time, this imbalance gradually decreases, approaching near-normal levels of approximately 10% by week 12, indicating an improvement in weight distribution between the legs.

**Figure 3:**
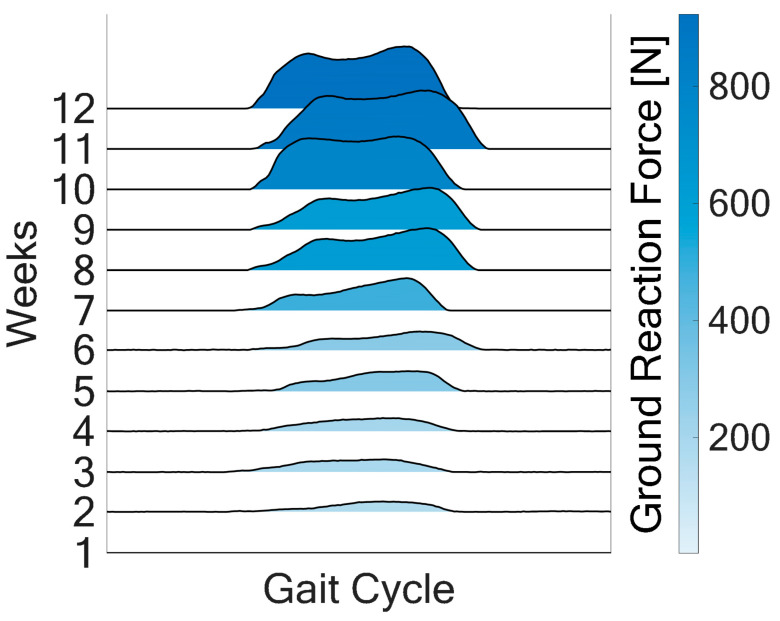
Illustration of the evolution of the ground reaction force in the right leg during a single gait cycle for the first seven weeks.

The shading of the curves in Figure 3 represents the magnitude of the ground reaction force, with darker shades indicating higher forces. The gradual darkening from earlier to later weeks highlights the progressive increase in weight-bearing capacity. In the first three weeks, the ground reaction force remains low and demonstrates a flattened curve, indicating a reduced ability to generate force during the stance phase of gait. In week 5, a more defined force curve emerges for the first time. This corresponds with increased limb stability and improved muscle engagement during the stance. The distinct peak indicates the restoration of better load transfer through the leg during the stance phase. After week 5, development of the two required maxima during the stance phase becomes visible, indicating a typical sign of an increasing physiological gait pattern.

**Figure 4:**
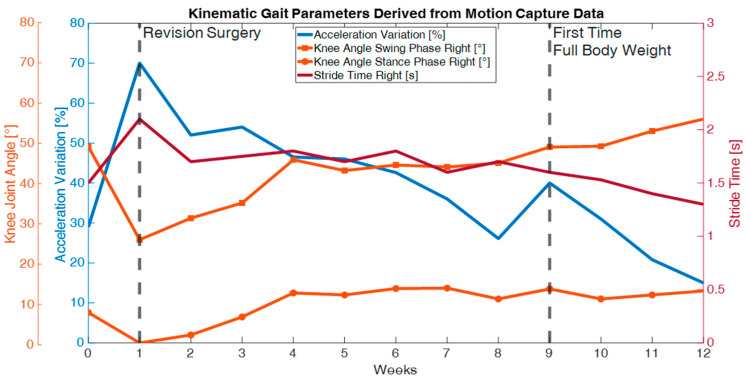
Temporal progression of kinematic parameters over a 12-week recovery period following a revision surgery.

Figure 4 shows the difference in acceleration between the right and left sides during walking. On day 2 after revision surgery, this difference peaks at nearly 70 percent. The patient’s initial attempts at walking led to this significant discrepancy. A fluid gait pattern with crutches is not yet achievable at this early stage. However, it is encouraging to note that the ratio between the two sides becomes increasingly similar as the healing progresses over time. By week nine, there was obvious further improvement. The patient walked without crutches for the first time, making him more cautious and slower when using the fractured leg than the uninjured leg. Notably, gait performance was worse before the revision operation than 12 weeks after the surgery. This suggests that at the time of the measurement taken four months after the first operation, the patient still experienced significant gait disturbances and dragging of the fractured leg, which in turn corresponded to the pain described by the patient before revision surgery. Treatment with an intramedullary nail considerably reduced these issues. The swing time of the right leg directly reflects this observation. The analysis of acceleration differences supports this finding. When examining the knee joint angle, flexion during the stance phase begins at 0 degrees and reaches only 10 degrees by the end of the fourth week. Pain-adapted walking during the first week eliminated the stance phase, preventing the initial maximum of the knee joint curve from appearing during each gait cycle.

## 5. Discussion

The data effectively captures a patient’s recovery process after severe healing disturbances with implant failure requiring revision surgery in a young patient suffering from a lower leg fracture. Initial post-surgery asymmetry and reduced function gradually improved over time as seen in the temporal trends and gait cycle curves. There was noticeable asymmetry and reduced function initially, but these gradually improved over time as demonstrated by both the temporal trends and gait cycle curves. This corresponded to the clinical and radiological events in the medical follow-up visits.

Conventional rehabilitation monitoring relies on subjective clinical assessments and scheduled radiographic imaging, typically capturing information at fixed intervals—for instance, using X-rays to evaluate a tibial fracture at 6 weeks, 12 weeks, and 18 months. However, validation of X-ray analysis is insufficient until approximately 8 or 12 weeks after the operation, leaving a diagnostic window during which the acute healing status is not monitored. In contrast, integrating sensor insoles and inertial motion capture enables objective, continuous, and quantitative evaluation of patient mobility. Recent studies have demonstrated the efficacy of wearable inertial sensors in rehabilitation settings. For instance, a study focusing on Parkinson’s disease patients utilized wearable sensors to identify early and subtle gait changes, which could serve as potential objective gait biomarkers for early diagnosis [20].

The study demonstrates for the first time several interesting parameters to monitor the course of bone healing. The primary goal is to use simple tools and parameters to accurately record changes in gait patterns during the healing process. The main advantage of the systems used is their independence from a special laboratory room. Both systems provide real-time feedback and function in both indoor and outdoor environments. Therefore, they offer great flexibility. This approach is supported by earlier work demonstrating that gait-related parameters, such as cadence and ground reaction forces (GRFs), are reliable indicators of musculoskeletal function and recovery [21,22]. The values obtained before the fractured leg reaches full weight-bearing status are particularly valuable. The factors of imbalance, ground reaction force, and acceleration variation, alone or in combination, may be helpful tools for monitoring the healing course using an additional, radiation-free tool. Therefore, they might be of interest in clinical practice and surgical aftercare. A large-scale study involving more subjects should validate the introduced parameters and establish precise reference values to identify a positive healing process or detect early signs of improper healing. Wearable technologies have already shown clinical utility in orthopedic rehabilitation and post-surgical monitoring [23,24,25]. Accordingly, the investigated parameters herein may be helpful additional parameters to evaluate the clinical course of events to estimate a healing disorder or—even more importantly—prevent the formation of a non-union and its accompanying high socio-economic burden [26,27,28]. We can simplify the setup by selecting specific parameters from the motion-capturing system. For instance, patients could sit on measuring insoles with one acceleration sensor on each thigh to monitor their movements. In the future, we anticipate a shift toward digital mobility outcomes, enabling patients to track their progress using data collected from mobile phones, smartwatches, and other digital devices. Ideally, they would also provide baseline measurements from before the fracture occurred. However, these initial results should serve as a foundational step in breaking down the healing process into its components, enabling precise monitoring of each patient. These systems provide instant feedback, which is essential for effective rehabilitation monitoring and compliance tracking. Unlike conventional methods, this approach operates independently of controlled environments, allowing for home-based and more real-time monitoring of continuous assessments. Additionally, this data analysis approach is not limited to lower extremity fractures. It can also be applied to kinematic evaluations of upper extremities and fracture healing trends, broadening its clinical applications.

## 6. Limitations

Motion capture systems and sensor technologies provide valuable insights into patient recovery but also have limitations. Problems with accuracy and reliability can arise due to measurement errors, noise, or drift over time, which can impact data accuracy. During measurements, personnel trained to operate the system must be present to verify its correct functioning. This means that independent continuous measurement by patients is not yet possible. Another challenge is the cost and accessibility of these systems. The purchase of hardware, additional software, and license costs is often beyond the means of many healthcare providers, especially for smaller clinics or regions with limited financial resources. The hardware of the Xsens Awinda system is not particularly expensive. As with many other purchased solutions, the software license is costly. Nevertheless, the system as a whole is well below the cost of a camera-based motion-capturing system. One can make a similar comparison for the measuring soles. The cost of several pairs of soles (in different sizes, for example), including the software package, is within the price range of a standard force plate. Both systems have a long service life. We have been using both measuring systems for several years and for various types of measurements (gait analyses, gym measurements, open terrain measurements, etc.).

For long-term measurements involving multiple patients or general introduction into routine practice, the amount of data generated by motion capture and sensor-based systems necessitates the use of neural networks or artificial intelligence algorithms for efficient processing, interpretation, and analysis. Without automation, the workload increases, making real-time assessments impractical.

The findings are based on a single patient, restricting the generalizability of the results. Future studies should involve larger and more heterogeneous cohorts to validate the robustness of the observed parameters. The study observation period was limited to 12 weeks; extending follow-up to 6–12 months could yield additional insights into long-term rehabilitation trajectories. This study serves as a pilot and feasibility study to conduct a study with a larger cohort and validate the results.

## 7. Conclusions

The patient’s kinetic and kinematic gait analysis reveals changes in biomechanical parameters during rehabilitation. During the first eight weeks of the partial weight-bearing phase, it is typical for the examined values to show greater asymmetry as the affected right side is still being spared. This asymmetry decreases with the transition to full weight-bearing from week 9 onwards, and normalization occurs more gradually over time. Adaptive compensation mechanisms can be observed during partial weight-bearing, which then decreases with the transition to full weight-bearing. Close observation of trends in the data can provide insight into whether the patient is adapting biomechanically to the increasing load or if there is deterioration, which would necessitate a change in rehabilitation measures. All in all, these are easy-to-use tools that do not require extensive prior knowledge and have a straightforward evaluation process, leading to quick findings.

## Figures and Tables

**Table 1:** Summary of conducted measurements and prescribed partial weight-bearing.

	Date	Location	Measured Activity	Recommended Partial Weight-Bearing	Further Information
M00	22 November 2023	Fitness Studio	Walking	Full Bodyweight	Preoperative
M01	22 December 2023	Hospital	Gait school/Rehabilitation exercise	0 kg	2 days postoperative
M02	29 December 2023	Hospital	Walking	20 kg	
M03	5 January 2024	At home	Measurement workflow	20 kg	
M04	12 January 2024	At home	Measurement Workflow	20 kg	
M05	19 January 2024	At home	Measurement workflow	20 kg	
M06	26 January 2024	At home	Measurement workflow	20 kg	
M07	31 January 2024	Hospital	Measurement workflow	45 kg	Follow-up appointment
M08	9 February 2024	At Home	Measurement workflow Walking without crutches Walking with one crutch Indoor cycling	45 kg	First-time full weight-bearing
M09	16 February 2024	At Home	Measurement workflow without crutches	Full Bodyweight	
M10	23 February 2024	At Home	Measurement workflow without crutches		
M11	12 March 2024	Hospital			Follow-up appointment

## Data Availability

The original contributions presented in the study are included in the article; further inquiries can be directed to the corresponding author. Researchers who wish to request access to data should send an email indicating the research purpose. Every request must be reviewed by the responsible institutional review boards, considering the risk of patient reidentification and compliance with applicable data protection rules.

## Supplementary Materials

The following supporting information can be downloaded at https://www.mdpi.com/article/10.3390/s25133853/s1: Figure S1: Moticon^TM^ sensor insole (A), distribution of pressure sensors and position of the coordinate system (B), and live display of pressure distribution while standing (C). Figure S2: Motion capturing system Xsens^TM^ setup (A), avatar in the Xsens^TM^ software (B), close-up of an IMU motion tracker (C), and a tracker with a strap (D). Figure S3: Comparison of the angles of the left and right knee joint during the measurements in week 2 and week 9. Figure S4: Comparison of the angles of the left and right ground reaction force during the measurements in week 2 and week 9.

## References

[1] Prasanth H., Caban M., Keller U., Courtine G., Ijspeert A., Vallery H., von Zitzewitz J. (2021). Weareble Sensor-Based Real-Time Gait Detection: A Systematic Review. Sensors.

[2] Warmerdam E., Orth M., Pohlemann T., Ganse B. (2023). Gait Analysis to Monitor Fracture Healing of the Lower Leg. Bioengineering.

[3] North K., Simpson G.M., Stuart A.R., Kubiak E.N., Petelenz T.J., Hitchcock R.W., Rothberg D.L., Cizik A.M. (2023). Early postoperative step count and walking time have greater impact on lower limb fracture outcomes than load-bearing metrics. Injury.

[4] Pfeufer D., Becker C.A., Faust L., Keppler A.M., Stagg M., Kammerlander C., Böcker W., Neuerburg C. (2020). Load-bearing detection with insole-force sensors provides new treatment insights in fragility fractures of the pelvis. J. Clin. Med..

[5] Saathvik A. (2021). Boompelli and Sambit Bhattacharya. Design of a telemetric gait analysis insole and 1-D convolutional neural network to track postoperative fracture rehabilitation. Proceedings of the LifeTech 2021–2021 IEEE 3rd Global Conference on Life Sciences and Technologies.

[6] Marmor M.T., Grimm B., Hanflik A.M., Richter P.H., Sivananthan S., Yarboro S.R., Braun B.J. (2022). Use of Wearable Technology to Measure Activity in Orthopaedic Trauma Patients: A Systematic Review. Indian J. Orthop..

[7] Braun B.J., Veith N.T., Rollmann M., Orth M., Fritz T., Herath S.C., Holstein J.H., Pohlemann T. (2017). Weight-bearing recommendations after operative fracture treatment—Fact or fiction? Gait results with and feasibility of a dynamic, continuous pedobarography insole. Int. Orthop..

[8] Braun B.J., Histing T., Herath S.C., Rollmann M.F.R., Reumann M., Menger M.M., Springer F., Andres A., Diebels S., Roland M. (2022). Bewegungsanalyse und muskuloskeletale Simulation in der Pseudarthrosentherapie—Erfahrungen und erste klinische Ergebnisse. Die Unfallchirurgie.

[9] Orth M., Ganse B., Andres A., Wickert K., Warmerdam E., Müller M., Diebels S., Roland M., Pohlemann T. (2023). Simulation-based prediction of bone healing and treatment recommendations for lower leg fractures: Effects of motion, weight-bearing and fibular mechanics. Front. Bioeng. Biotechnol..

[10] Warmerdam E., Wolff C., Orth M., Pohlemann T., Ganse B. (2024). Long-term continuous instrumented insole-based gait analyses in daily life have advantages over longitudinal gait analyses in the lab to monitor healing of tibial fractures. Front. Bioeng. Biotechnol..

[11] Horenstein R.E., Goudeau Y.R., Lewis C.L., Shefelbine S.J. (2020). Using magneto-inertial measurement units to pervasively measure hip joint motion during sports. Sensors.

[12] Horenstein R.E., Lewis C.L., Yan S., Halverstadt A., Shefelbine S.J. (2019). Validation of magneto-inertial measuring units for measuring hip joint angles. J. Biomech..

[13] Ibrahim N.I., Ahmad M.S., Zulfarina M.S., Zaris S.N.A.S.M., Mohamed I.N., Mohamed N., Mokhtar S.A., Shuid A.N. (2018). Activities of daily living and determinant factors among older adult subjects with lower body fracture after discharge from hospital: A prospective study. Int. J. Environ. Res. Public Health.

[14] Bovi G., Rabuffetti M., Mazzoleni P., Ferrarin M. (2011). A multiple-task gait analysis approach: Kinematic, kinetic and EMG reference data for healthy young and adult subjects. Gait Posture.

[15] Lauenroth A., Laudner K., Schulze S., Delank K.S., Fieseler G., Schwesig R. (2018). Laufbandbasierte Gangreferenzdaten für gesunde Probanden: Abhängigkeit von funktionellen und morphologischen Parametern. Man. Med..

[16] Marimon X., Mengual I., López-de-Celis C., Portela A., Rodríguez-Sanz J., Herráez I.A., Pérez-Bellmunt A. (2024). Kinematic Analysis of Human Gait in Healthy Young Adults Using IMU Sensors: Exploring Relevant Machine Learning Features for Clinical Applications. Bioengineering.

[17] Medved V., Vastola R., Albano D., Pećina M. (2022). Gait Analysis. Measurement and Analysis of Human Locomotion.

[18] Hoffmann T., Falz R., Busse M. (2014). Winkelveränderungen verschiedener Gelenke der unteren Extremitäten bei unterschiedlichen Geschwindigkeiten und Neigungswinkeln. Klin. Sport. Sport. Med..

[19] Newman S.D.S., Mauffrey C.P.C., Krikler S. (2011). Distal metadiaphyseal tibial fractures. Injury.

[20] Zhang W., Ling Y., Chen Z., Ren K., Chen S., Huang P., Tan Y. (2024). Wearable sensor-based quantitative gait analysis in Parkinson’s disease patients with different motor subtypes. npj Digit. Med..

[21] Kaufman K.R., Hughes C., Morrey B.F., Morrey M., An K.N. (2001). Gait characteristics of patients with knee osteoarthritis. J. Biomech..

[22] Perry J., Burnfield J. (2010). Gait Analysis: Normal and Pathological Function.

[23] Amin T., Mobbs R.J., Mostafa N., Sy L.W., Choy W.J. (2021). Wearable devices for patient monitoring in the early postoperative period: A literature review. mHealth.

[24] Kobsar D., Masood Z., Khan H., Khalil N., Kiwan M.Y., Ridd S., Tobis M. (2020). Wearable inertial sensors for gait analysis in adults with osteoarthritis—A scoping review. Sensors.

[25] Muro-de-la-Herran A., García-Zapirain B., Méndez-Zorrilla A. (2014). Gait analysis methods: An overview of wearable and non-wearable systems, highlighting clinical applications. Sensors.

[26] Antonova E., Le T.K., Burge R., Mershon J. (2013). Tibia shaft fractures: Costly burden of nonunions. BMC Musculoskelet. Disord..

[27] Court-Brown C.M., Caesar B. (2006). Epidemiology of adult fractures: A review. Injury.

[28] Rupp M., Biehl C., Budak M., Thormann U., Heiss C., Alt V. (2018). Diaphyseal long bone nonunions—Types, aetiology, economics, and treatment recommendations. Int. Orthop..

