# Supplementary material for: From Injury to Full Recovery: Monitoring Patient Progress Through Advanced Sensor and Motion Capture Technology
Source: Sensors (Basel). 2025 Jun 20;25(13):3853. doi: 10.3390/s25133853 (PMC12251791; doi:10.3390/s25133853)
Supplement: Supplementary file 1 [file sensors-25-03853-s001.zip › sensors-3568979-supplementary.pdf]

sensors

MDPI

Supplementary Material

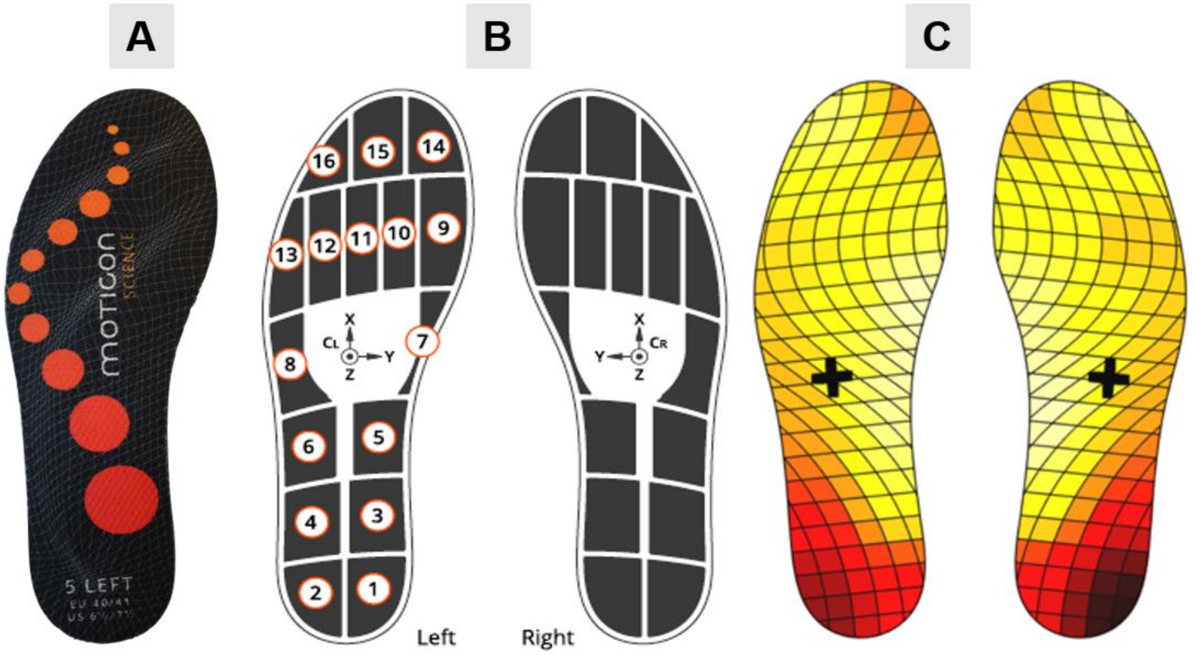

Figure S1. Moticon™ sensor insole (A), Distribution of pressure sensors and position of the coordinate system (B), Live display of pressure distribution while standing (C).

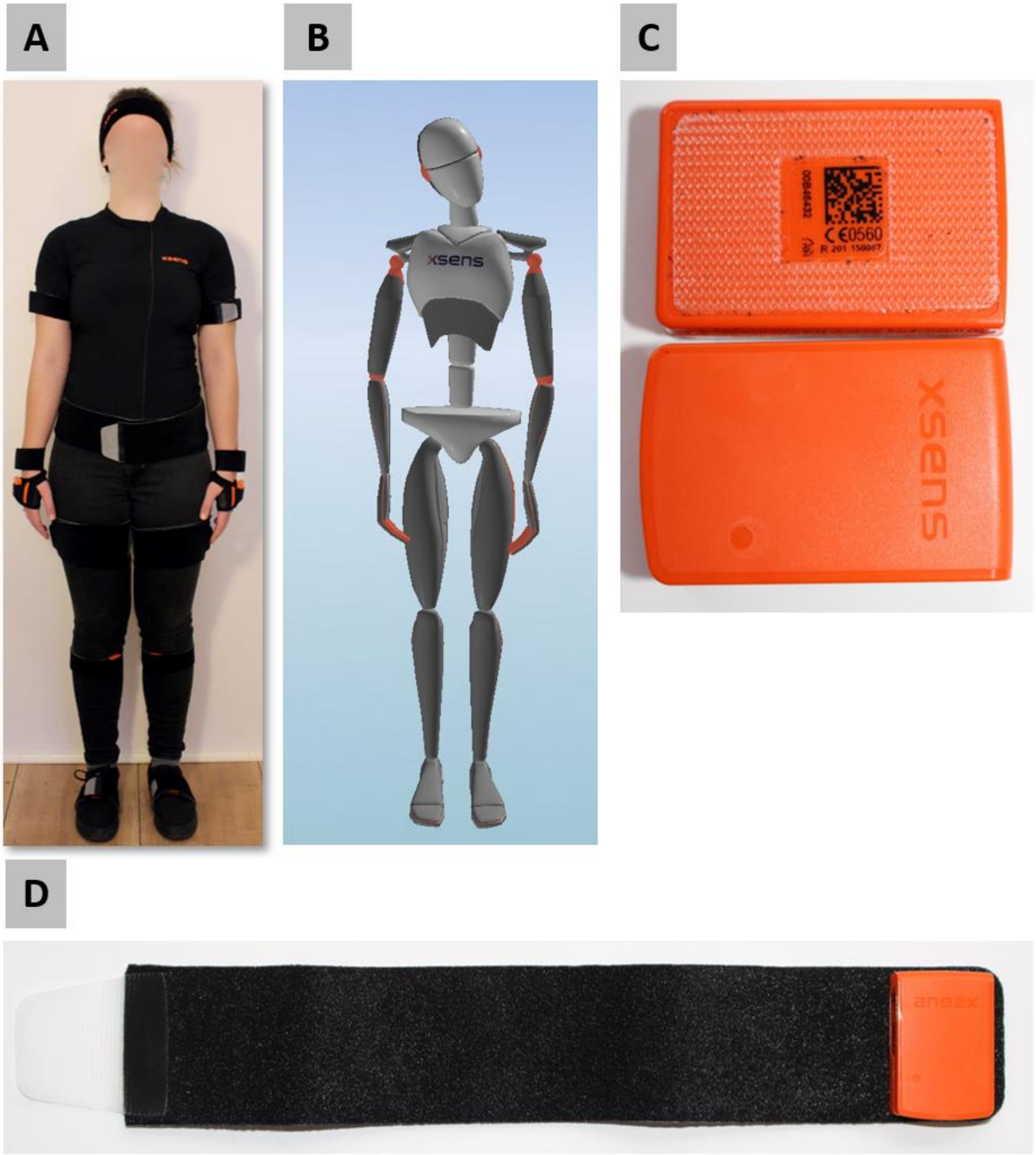

Figure S2. Motion capturing system Xsens™ setup (A), Avatar in the Xsens™ software (B), close up of an IMU motion tracker (C), and tracker with strap (D).

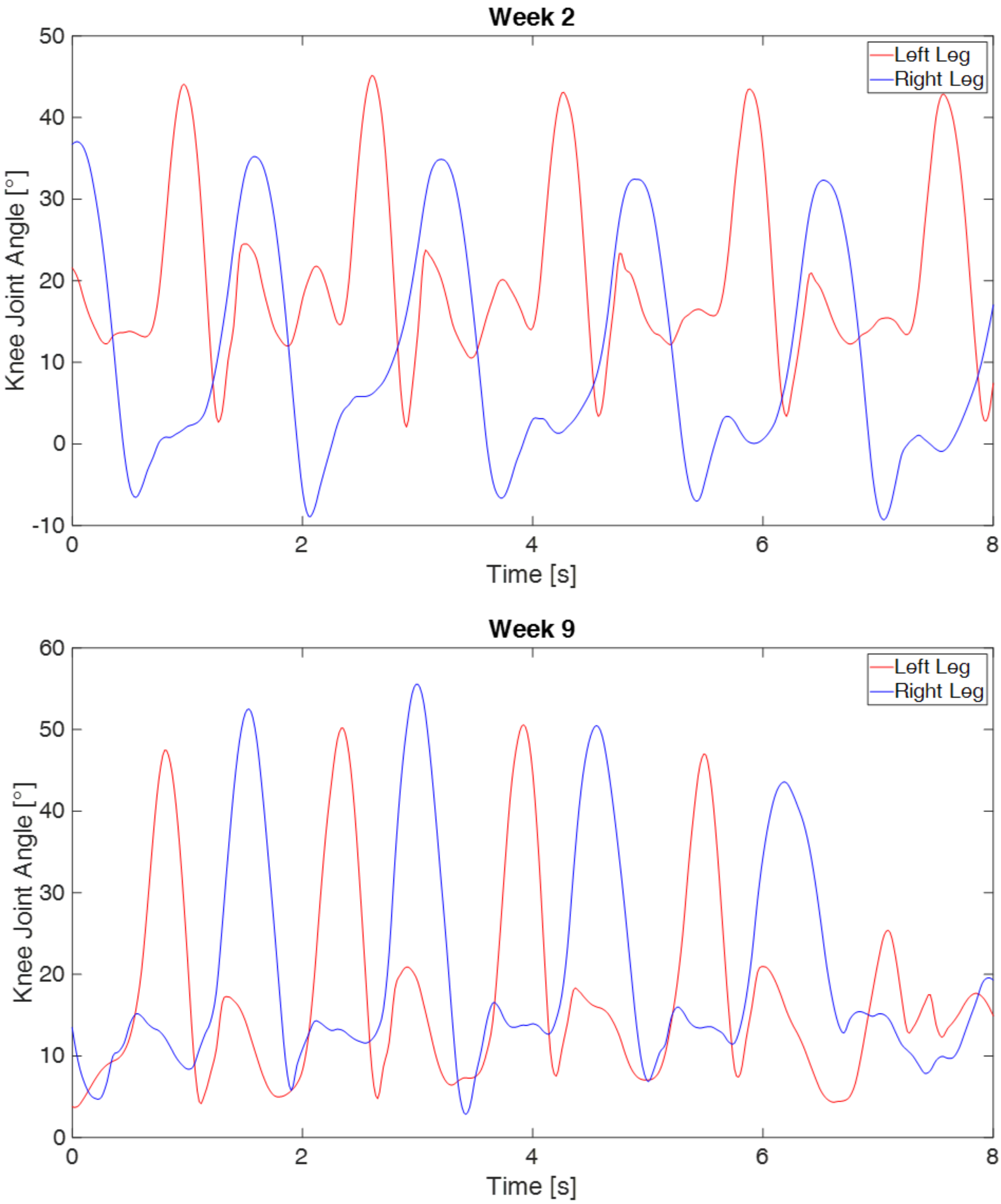

Figure S3. Comparison of the angles of the left and right knee joint during the measurements in week 2 and week 9.

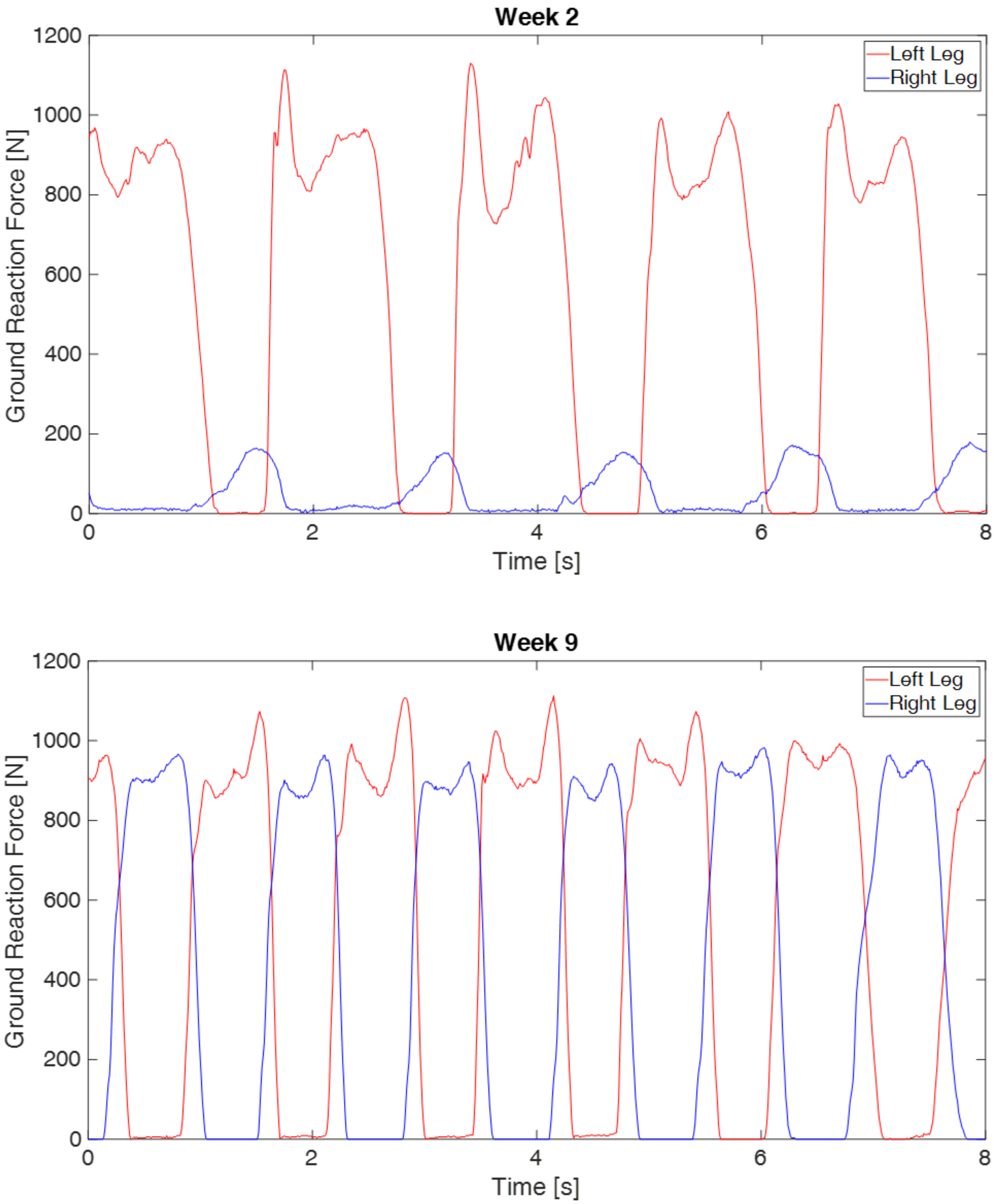

Figure S4. Comparison of the angles of the left and right ground reaction force during the measurements in week 2 and week 9.
